# Converging Molecular Mechanisms of Nucleated Cell Death Pathways and Procoagulant Platelet Formation

**DOI:** 10.3390/cells14141075

**Published:** 2025-07-14

**Authors:** Cong Li, Attila Braun, Juan Zu, Thomas Gudermann, Elmina Mammadova-Bach, Hans-Joachim Anders

**Affiliations:** 1 Division of Nephrology, Department of Medicine IV, Ludwig Maximilian University Hospital, 80336 Munich, Germany; cong.li@med.uni-muenchen.de; 2 Walther Straub Institute of Pharmacology and Toxicology, Ludwig Maximilian University, 80336 Munich, Germany; attila.braun@lrz.uni-muenchen.de (A.B.); thomas.gudermann@lrz.uni-muenchen.de (T.G.); 3 Center for Neuropathology, Ludwig Maximilian University, 81377 Munich, Germany; juan.zu@med.uni-muenchen.de; 4 German Center for Lung Research (DZL), Munich, Germany

**Keywords:** cell death, platelet procoagulant activity, apoptosis, necrosis, necroptosis, pyroptosis, ferroptosis, thrombosis, coagulation

## Abstract

Procoagulant platelets are a specialized subset of activated platelets that externalize phosphatidylserine (PS) on their surface, facilitating the assembly of tenase and prothrombinase complexes and enhancing thrombin generation and clot formation. Although procoagulant platelet formation shares certain features with nucleated cell death pathways, such as mitochondrial dysfunction, calcium (Ca^2+^) overload, membrane blebbing, and microvesiculation, it differs in key molecular mechanisms, notably lacking nuclei and caspase-dependent deoxyribonucleic acid (DNA) fragmentation. Interestingly, molecular components of nucleated cell death pathways in platelets can promote thrombus formation without impacting platelet lifespan. Under pathological conditions, excessive platelet activation may result in platelet lysis, resembling the complete activation of nucleated cell death pathways and contribute to thrombocytopenia. This review compares procoagulant platelet formation with various nucleated cell death pathways, including necrosis, necroptosis, pyroptosis, and ferroptosis, and explores their role in pathological thrombosis and blood clotting. A deeper understanding of mechanisms may help in developing targeted therapies to prevent aberrant blood clotting, platelet death and thrombocytopenia.

## 1. Introduction

Cell death is a vital process for tissue homeostasis, immune regulation, and the elimination of damaged or dangerous cells. In nucleated cells, death can occur through multiple distinct pathways, which differ in their molecular mechanisms, morphological features, immunological consequences, and susceptibility to regulation [1]. These pathways can broadly be classified into accidental and regulated forms of cell death, with further subdivisions based on specific biochemical and functional characteristics [1]. Regulated forms of cell death such as apoptosis, necroptosis, pyroptosis, and ferroptosis are distinct in their triggers and execution mechanisms, while accidental cell death occurs in response to extreme stress [1,2,3]. Regulated cell death is a genetically encoded process that can be modulated by molecular signals or pharmacological intervention. These pathways are tightly controlled and often play essential roles in development, disease, and immune responses. Immunogenic cell death represents a unique intersection of regulated death and immune activation, highlighting the functional diversity of how cells die and the consequences for tissue and organismal health [3].

Although platelets are anucleate cell fragments derived from megakaryocytes (MKs) [4], they possess a complex intracellular machinery capable of sensing stress, executing death programs, and releasing signals that influence immune and vascular responses [5]. Platelets engage in tightly regulated cell death processes, which are crucial for hemostasis, thrombosis, inflammation, and platelet clearance. Platelet death mechanisms are recognized based on morphological, biochemical, and functional changes in activated and aging platelets [6]. During aging, platelets accumulate mitochondrial damage and exhibit reduced adenosine triphosphate (ATP) production, which impairs the function of calcium (Ca^2+^) pumps such as the plasma membrane Ca^2+^-ATPase and the sarcoplasmic/endoplasmic reticulum Ca^2+^ ATPase [7]. This dysfunction results in cytosolic Ca^2+^ overload, subsequently activating scramblase enzymes that translocate phosphatidylserine (PS) from the inner to the outer leaflet of the plasma membrane [7]. Under physiological conditions, membrane asymmetry is maintained by flippases, which remove PS from the outer leaflet. However, in both apoptotic nucleated cells and activated platelets, scramblase activity increases, while flippase activity diminishes, leading to sustained PS exposure [8]. In platelets, this shift contributes to procoagulant activity, whereas in nucleated cells, it marks the initiation of programmed cell death. In addition, aging platelets undergo desialylation, a process in which terminal sialic acid residues are removed from platelet surface glycoproteins [8]. This desialylation further disrupts membrane asymmetry, promotes PS exposure, and targets platelets for clearance by macrophages within the reticuloendothelial system [8].

Upon activation, platelets display both functional and morphological heterogeneity, forming two distinct subpopulations: aggregatory and procoagulant platelets [9]. Aggregatory platelets mediate adhesion and aggregation, forming the initial platelet plug, whereas procoagulant platelets enhance thrombin generation and drive the coagulation cascade [9]. By linking primary and secondary hemostasis, procoagulant platelets regulate clot formation and thrombus growth [9]. Morphologically, aggregatory platelets extend pseudopodia, while procoagulant platelets adopt a balloon-like morphology characterized by extensive membrane blebbing which facilitates PS exposure and supports the assembly of tenase and prothrombinase complexes [10]. Both types of platelets release bioactive mediators that promote further platelet recruitment and immune cell activation at sites of vascular injury [11].

Procoagulant platelets exhibit a distinct pro-inflammatory phenotype, releasing effectors such as inorganic polyphosphate and cytokines. Procoagulant platelet formation shares several hallmarks with nucleated cell death pathways, including mitochondrial dysfunction, reactive oxygen species (ROS) generation, cytosolic Ca^2+^ overload, membrane blebbing and microvesicle shedding, ultimately resulting in the loss of membrane integrity [9]. Ballooned platelets are associated with the terminal stages of platelet activation, sharing many similarities with nucleated cell death pathways. However, in many pathological contexts, activated platelets remain viable despite displaying several characteristics associated with cell death pathways [12]. These include mitochondrial dysfunction, ROS production, Ca^2+^ deregulation and membrane remodeling, without progressing to complete apoptosis. In severe pathological conditions, however, platelets may undergo complete cell lysis, closely mimicking the morphological and biochemical features of nucleated cell death [12].

This review discusses the principle of nucleated cell death pathways—apoptosis, necrosis, necroptosis, pyroptosis, and ferroptosis—and compares their molecular components with pathomechanisms triggering procoagulant platelet formation. Furthermore, we assess their potential contributions to pathological thrombosis and blood clot formation and discuss how these shared molecular pathways may help in the development of targeted antiplatelet therapies. This comparative analysis may stimulate researchers to further investigate selective regulators of cell death-associated mechanisms in MKs and platelets, offering novel strategies to prevent abnormal platelet production, thrombus formation, and blood clotting.

## 2. Apoptosis Pathways in Nucleated Cells Versus Platelets

Programmed cell death is a highly regulated process, classically divided into receptor-mediated extrinsic and non-receptor-mediated intrinsic apoptotic pathways in nucleated cells. These pathways activate proteolytic enzymes known as caspases through distinct mechanisms, ultimately leading to cell death [13].

### 2.1. Extrinsic and Intrinsic Apoptosis Pathway Activation Mechanisms

The extrinsic pathway is initiated by signals, typically mediated by the binding of extracellular death ligands to specific death receptors such as FAS (CD95: cluster of differentiation 95), tumor necrosis factor receptor (TNFR), or TNF-related apoptosis-inducing ligand (TRAIL) receptors (DR4 and DR5: death receptors 4 and 5) on the cell surface [14]. Ligand binding induces receptor oligomerization and the recruitment of adaptor proteins, such as FADD (Fas-associated death domain), which subsequently recruit and activate initiator caspase-8 [14]. Activated caspase-8 directly cleaves and activates downstream executor caspases, including caspases-3, -6, and -7, driving the biochemical and morphological changes associated with apoptosis [14]. In addition, caspase-8 can cleave BH3 interacting-domain death agonist (BID), a pro-apoptotic member of the B-cell lymphoma 2 (BCL-2) protein family, thereby linking the extrinsic pathway to the intrinsic (mitochondrial) pathway and promoting mitochondrial damage [1]. The intrinsic apoptotic pathway in nucleated cells is triggered by intracellular stress factors that activate pro-apoptotic proteins (BCL-2-associated X protein: BAX and BCL-2 homologous antagonist/killer: BAK), forming pores in the outer mitochondrial membrane. This leads to the release of Cytochrome C, second mitochondria-derived activator of caspases (SMAC), and apoptosis-inducing factor (AIF) into the cytoplasm, leading to deoxyribonucleic acid (DNA) fragmentation, chromatin condensation, membrane blebbing, and PS exposure. These events generate an “eat-me” signal, promoting phagocytic clearance [13]. Anti-apoptotic proteins, including B-cell lymphoma 2 (BCL-2) and B-cell lymphoma-extra large (BCL-XL), inhibit BAX/BAK activation, preserve membrane integrity and block apoptosis. However, mitochondrial damage induced by BH3-only proteins can bypass this protection, irreversibly committing the cell to apoptosis [15].

### 2.2. Intrinsic Apoptosis Pathway Activation Mechanisms in MKs and Platelets

Although the molecular components of the extrinsic apoptotic pathway exist in MKs and can trigger cell death, platelets do not express FAS and TRAIL receptors [16,17,18,19]. Therefore, these receptor-mediated pathways cannot be directly activated in platelets. However, activated platelets can expose the Fas ligand (FasL) on their surface, enabling them to induce FAS-receptor-induced cell death in neighboring cells [20]. Despite lacking a nucleus and death receptors, circulating platelets retain essential components of the apoptotic machinery. Protein expressions of BCL-family members, BH3-only proteins, including Bcl-2-associated death promoter (BAD), BCL-2-interacting killer (BIK), and BCL-2-interacting mediator of cell death (BIM), or BAX and BAK are detectable in platelets [19]. Additionally, downstream effectors of death receptor signaling, such as FADD, BID, and caspases are expressed in platelets [17]. Interestingly, caspase-3, 7, 8, or 9 deficiencies do not impair platelet count in mice, indicating that the extrinsic pathway does not regulate platelet production [17].

In sharp contrast, the intrinsic apoptotic pathway dominates this process, regulating MK and platelet survival. Genetic ablation of the floxed *Bclx* gene using the MMTV-LTR-Cre in the hematopoietic system induces splenomegaly, hemolytic anemia, and thrombocytopenia in mice [21]. Similarly, *Bclx* gene deletion in MKs using a PF4-Cre system results in a marked reduction in platelet count, leading to severe thrombocytopenia, which is due to increased MK apoptosis and impaired platelet shedding [22]. A decrease in BCL-XL levels over time triggers BAK activation, leading to platelet clearance by macrophages in the reticuloendothelial system. Pharmacological inhibition of BCL-XL with agents such as ABT-737 or navitoclax (ABT-263) induces platelet death, characterized by hallmarks of apoptosis observed in nucleated cells, including mitochondrial damage, caspase activation, Ca^2+^ overload, and PS exposure [23,24,25]. On the other hand, genetic deletion of *Bak* and *Bax* confers platelet protection against cell death [22]. BAK deficiency in mice alone extends platelet lifespan, similar to BAK/BAX double deficiency, indicating a dominant role for BAK [22,26]. The BH3-only protein mimetic ABT-737 could effectively induce mitochondrial damage, caspase activation, and cell death in wild-type MKs, while BAK/BAK-deficient MKs are protected from thrombocytopenia [22]. Consistent with this, mice lacking both BAK and BAX display a prolonged platelet lifespan [4,22]. Interestingly, MK-specific deletion of the *Bcl-2* gene in mice results in normal platelet count and lifespan, and the BCL-2 inhibitor ABT-199 does not alter platelet production in animal models or human patients [27]. These studies suggest that selective BCL-2 inhibition remains a promising therapeutic strategy in cancer without the risk of inducing thrombocytopenia. The loss of BCL-XL function completely disturbs the balance between pro- and anti-apoptotic signaling in MKs and other BCL family members cannot compensate for the lack of BCL-XL function [27].

In nucleated cells and platelets, apoptosis is regulated by the balance of activated pro- and anti-apoptotic proteins. In platelets, however, the absence of DNA and limited protein synthesis prevent transcriptional regulation, making apoptosis reliant on the availability and post-translational modification of proteins from MKs. Therefore, the ‘molecular clock’ model proposes that BCL-XL degrades more rapidly in platelets than pro-apoptotic proteins, ultimately triggering platelet death [25]. However, deletion of various pro-apoptotic genes in mice revealed that only BAD deficiency could moderately prolong platelet lifespan [28], indicating a complex regulatory crosstalk between pro- and anti-apoptotic pathways, likely driven by pathway dysregulation rather than absolute protein abundance.

Both activated and apoptotic platelets expose PS [24]. Treatment with the pro-apoptotic BH3 mimetic ABT-737 induces PS exposure, procoagulant activity and thrombin generation in platelets [23]. In this context, PS exposure is BAK/BAX- and caspase-dependent but unaffected by platelet antagonists or extracellular Ca^2+^ chelation [23]. Conversely, agonist-induced platelet PS exposure remains intact in BAK−/−BAX−/− or caspase inhibitor-treated platelets but is eliminated by platelet antagonists. Ca^2+^ chelators fail to inhibit PS exposure in apoptotic platelets, suggesting a dispensable role of Ca^2+^ channels and subsequent extracellular Ca^2+^ entry in platelet apoptosis [23]. In nucleated cells, Ca^2+^ mediates a full apoptotic program, culminating in nuclear, mitochondrial, and cytoplasmic degradation [29]. In sharp contrast, receptor-induced Ca^2+^ overload in platelets triggers only procoagulant activity without inducing cell death, reflecting their specialized anucleate nature [9,30]. The consequences of mitochondrial dysfunction also differ between apoptotic and procoagulant platelets. Platelet apoptosis is strongly BAK/BAX-dependent, involving Cytochrome C release and caspase activation, leading to PS exposure and membrane blebbing without alpha (α)-granule release [19]. In procoagulant platelets, mitochondrial dysfunction is Cyclophilin D (CypD)-dependent [31], and both PS exposure and α-granule release require Ca^2+^ [11,32]. These findings suggest that alternative molecular routes regulate PS exposure in platelets, partially separated from both receptor-mediated signaling and nucleated cell death mechanisms [23]. The genetic ablation of pro-apoptotic proteins does not impair platelet activation or agonist-induced PS exposure in mice, further highlighting the functional differences between procoagulant and apoptotic platelets [23].

**Figure 1:**
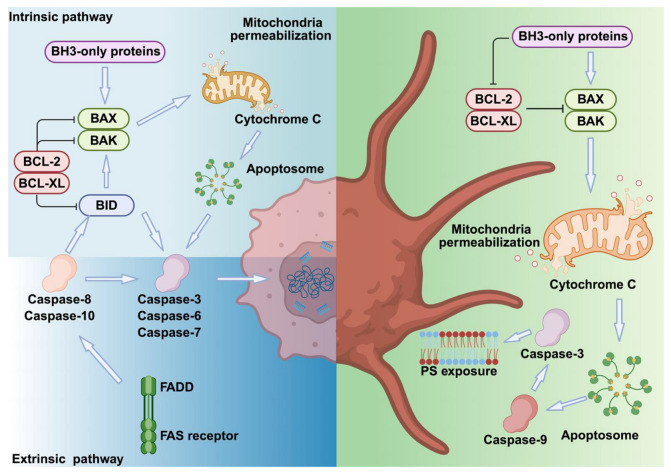
Comparison of apoptotic pathways in nucleated cells and platelets. In nucleated cells, death receptor activation recruits adaptor proteins like FADD to form the death-inducing signaling complex (DISC), activating caspase-8. This triggers executioner caspases (caspase-3, -6, -7) and cleaves BID, linking extrinsic and intrinsic pathways. BAX and BAK form mitochondrial pores, releasing cytochrome C, activating the apoptosome (caspase-9), and leading to DNA fragmentation, membrane blebbing, and PS exposure. BCL-2 and BCL-XL inhibit BAX/BAK, while BH3-only proteins promote apoptosis. Platelets share apoptotic machinery but lack nuclei and death receptors, preventing full apoptosis. They display membrane blebbing, PS exposure, and microvesiculation while retaining procoagulant function. In platelets, Ca^2+^ overload triggers procoagulant PS exposure independently of BAX/BAK and caspases, whereas apoptotic PS exposure is caspase- and BAX/BAK-dependent but Ca^2+^-independent. These differences reflect unique platelet adaptations for hemostasis.

In summary, in nucleated cells, apoptosis occurs via extrinsic (receptor-mediated) and intrinsic (mitochondrial) pathways. Platelets lack death receptors and rely mainly on the intrinsic pathway, regulated by BCL-XL and BAK. Loss of BCL-XL triggers BAK-dependent apoptosis, while BCL-2 plays a redundant role. Platelet apoptosis follows a ‘molecular clock’ as BCL-XL degrades. PS exposure marks both apoptotic and procoagulant platelets, but through distinct mechanisms, reflecting specialized regulation compared to nucleated cells (Figure 1, Table 1).

## 3. Necrosis in Nucleated Cells Versus Platelets

### 3.1. Mitochondrial Dysfunction as a Trigger for Necrosis in Nucleated Cells

In nucleated cells, necrosis is often triggered by pathological stress factors such as ischemia, trauma, or infection. While apoptotic cells preserve plasma membrane integrity, to avoid provoking unwanted immune responses, necrotic cells rupture their membranes, releasing intracellular contents, including damage-associated molecular patterns (DAMPs) [18]. This cell lysis initiates a robust inflammatory response, recruiting immune cells to the necrotic area [18]. Necrosis is triggered by the formation of the mitochondrial permeability transition pore (MPTP), regulated by several proteins, including CypD, adenine nucleotide translocator (ANT), voltage-dependent anion channel (VDAC), oligomycin-sensitive binding protein (OSCP) and mitochondrial inorganic phosphate carrier (PiC) [33].

CypD deficiency prevents MPTP formation and abolishes Ca^2+^- and ROS-dependent necrotic cell death without affecting apoptosis [34,35]. Elevated cytosolic Ca^2+^ levels trigger MPTP opening and subsequently collapsing mitochondrial membrane potential (Δψm) and ATP synthesis. This energy depletion disrupts metabolic pathways, leading to irregular Ca^2+^ transport across the mitochondrial and plasma membranes. Mitochondrial damage induces Ca^2+^ pump dysfunctions and subsequent Ca^2+^ overload in the cytoplasm, increasing ROS production, thereby enhancing Ca^2+^-dependent signaling and oxidative reactions [21]. Elevated ROS production, in turn, oxidizes lipids and proteins, thereby resulting in the subsequent leakage of Ca^2+^ store and lysosome membranes, further amplifying the process of necrosis [21]. Although dysregulated Ca^2+^ homeostasis is also involved in apoptosis, cytoplasmic Ca^2+^ levels remain lower in apoptotic cells than in necrotic cells, thereby protecting cells from complete cell lysis [22].

### 3.2. Procoagulant Activity as a Hallmark of Platelet Necrosis

Necrosis represents a distinct form of cell death characterized by its inflammatory nature, which differs significantly between nucleated cells and platelets. Lacking a nucleus, platelets do not release nuclear DAMPs (e.g., histones), which are key drivers of inflammation in nucleated cell necrosis. However, due to their abundance, necrotic platelets can still trigger significant inflammatory responses by releasing other DAMPs, such as mitochondrial DNA, as well as cytokines. Platelet necrosis shares several molecular components with nucleated cells, including MPTP formation, Ca^2+^ overload, ROS production, and loss of membrane integrity [9,11].

The mitochondrial Ca^2+^ uniporter (MCU) facilitates rapid mitochondrial Ca^2+^ influx in activated platelets, promoting CypD-induced MPTP formation, PS exposure, and procoagulant activity [23]. Although the CypD-induced necrotic pathway rapidly kills nucleated cells, in sharp contrast, activated platelets can tolerate CypD-dependent MPTP formation and Ca^2+^ overload without undergoing cell death [36]. Depending on the external stimuli, the antioxidant system including catalase, glutathione and superoxide dismutase (SOD) enzymes removes ROS and the Ca^2+^ pump can reduce cytoplasmic Ca^2+^ levels thereby preventing mitochondria dysfunction and platelet death.

Using genetic mouse models, both MCU- and CypD-deficient platelets display impaired MPTP formation, reduced PS exposure, and diminished thrombin generation, highlighting the importance of this nucleated cell death pathway in procoagulant platelet formation [9,37]. Interestingly, CypD-deficient mice do not exhibit a marked bleeding diathesis, and Ca^2+^ ionophores can fully revert PS exposure defects in mutant platelets. Furthermore, challenging CypD deficient mice using different in vivo arterial thrombosis mouse models showed controversial results [36,38,39]. This suggests that the importance of CypD-mediated signaling probably strongly depends on the experimental (pathological) conditions. Pharmacological inhibition of CypD-dependent MPTP formation using Cyclosporin A (CsA) reduces antigenic modulation of platelet integrin αIIbβ3, PS externalization, and high-level fibrinogen retention [36]. However, a higher concentration of CsA in the absence of a physiological platelet agonist induces PS exposure and lactate dehydrogenase release, indicating dose-dependent off-target effects of CsA on the platelet membrane [40]. Rapid PS exposure is highly regulated by increased cytoplasmic Ca^2+^ level, and this was attenuated by CsA treatment, independently of its effect on mitochondrial permeability [41]. Altogether, these findings suggest the existence of functional redundancy in platelet signaling pathways regulating necrosis and procoagulant activity. Necrotic platelets exhibit distinct morphological characteristics to apoptotic platelets, including the loss of intracellular organelles and granule content, degradation of the actin cytoskeleton, and the loss of their adherent or aggregatory functions [42]. These necrotic platelets primarily support coagulation, thrombin generation, and subsequent fibrin mesh formation. Increased platelet necrosis is frequently observed in disease conditions in which mechanical stress triggers cell death such as mechanical circulatory support or stenotic arteries [43]. High levels of ROS production in thrombotic diseases may also account for platelet necrosis [44]. Functional studies have demonstrated that the procoagulant response in CypD-deficient platelets is strongly reduced following stimulation with thrombin and convulxin, lacking the essential capacity to activate the prothrombinase complex [25].

Recently, procoagulant platelets have emerged as critical mediators in a variety of pathophysiological conditions. They prevent inflammatory bleeding by localizing coagulation to sites of vascular injury, a process dependent on CypD and the membrane scramblase TMEM16F, both critical mediators of platelet procoagulant activity. Platelet integrin αIIbβ3 outside-in and glycoprotein VI (GPVI) signaling pathways were shown to act synergistically to initiate this response, and the simultaneous inhibition of both pathways disrupted vascular integrity [45]. In mouse models, the platelet-specific deletion of CypD or TMEM16F protects against venous thrombosis. Similarly, pharmacological inhibition of platelet procoagulant activity with the carbonic anhydrase inhibitor methazolamide reduces thrombus formation without impairing trauma-induced hemostasis, highlighting its therapeutic potential [46]. Beyond their prothrombotic function, necrotic procoagulant platelets also play a critical role in immune cell recruitment by providing a surface that facilitates neutrophil accumulation, which exacerbates brain injury following ischemic stroke [47]. Procoagulant platelets also contribute to tumor progression by delivering immune checkpoint molecules that promote a protumorigenic myeloid response while suppressing antitumor lymphocyte activity [48].

**Figure 2:**
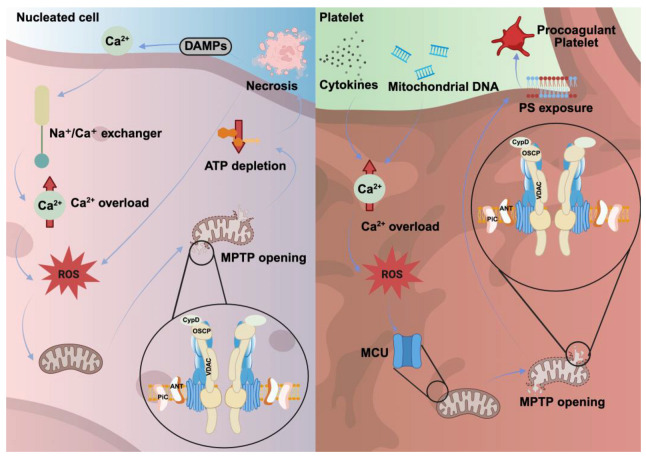
Necrosis in nucleated cells and platelets. In nucleated cells, ischemia, trauma, or infection lead to cell lysis, releasing DAMPs, which are accompanied by cytoplasmic Ca^2+^ overload and ROS production. This process culminates in mitochondrial damage and necrosis through the formation and opening of the MPTP, regulated by CypD, ANT, VDAC, OSCP and PiC. In platelets, elevated cytokines and extracellular mitochondrial DNA induce intracellular Ca^2+^ overload and increased ROS production. Subsequently, MCU facilitates rapid mitochondrial Ca^2+^ influx in activated platelets, promoting CypD-induced MPTP formation, PS exposure on the platelet surface, and enhanced platelet procoagulant activity.

In summary, necrosis in nucleated cells triggers inflammation through abundant cell death and DAMP release, while platelet necrosis primarily leads to PS exposure and the formation of highly activated procoagulant platelets with minimal inflammatory involvement and without full cell lysis. In extreme pathological conditions, necrotic platelets are ruptured thereby releasing DAMPs and promoting excessive blood clot formation and inflammation (Figure 2, Table 2).

## 4. Necroptosis Pathways in Nucleated Cells Versus Platelets

### 4.1. Necroptosis as a Controlled Alternative Death Pathway in Nucleated Cells

In nucleated cells, necroptosis plays a key role in immune defense, inflammation, and cancer, combining features of apoptosis and necrosis [49]. It becomes particularly important when apoptosis is inhibited, enabling the immune system to eliminate cells that might evade death [3]. Unlike the immune-silent apoptosis, necroptosis provokes inflammation through the release of intracellular components and DAMPs, resembling necrosis [49,50].

The necroptotic pathway is assembled by distinct molecular players. It is primarily initiated by death receptors, such as tumor necrosis factor receptor 1 (TNFR1) and toll-like receptors (TLRs) in response to ligands like TNF-α, FasL, and pathogen-associated molecular patterns (PAMPs) [49,50]. A hallmark of necroptosis is the involvement of receptor-interacting protein kinase (RIPK) family members and their downstream effector, mixed lineage kinase domain-like protein (MLKL) [49]. Upon activation of TNFR1 or TLR activation, RIPK1 phosphorylates RIPK3, which subsequently activates MLKL [49]. Activated MLKL oligomerizes and translocates to the plasma membrane, where it forms disruptive pores, releasing DAMPs and pro-inflammatory mediators, thereby triggering robust inflammatory responses and amplifying immune cell recruitment [50].

### 4.2. Necroptotic Platelets: Regulators of Hemostasis and Thrombosis

RIPK3 signaling regulates hemostasis and thrombosis [51]. Although MKs express molecular components of the necroptotic pathway, their role in cell death differs from that in nucleated cells [52]. While RIPK3 deficiency prevents MLKL phosphorylation and pore formation, neither RIPK3 nor MLKL deficiency affects platelet production in genetic mouse models, suggesting that RIPK3-mediated necroptosis plays a minor or redundant role in megakaryopoiesis [52]. However, a constitutively active MLKL variant (D139V mutation) significantly reduces platelet counts in knock-in mice, indicating that aberrant MLKL hyperactivation can induce thrombocytopenia, likely through necroptosis which is triggered by RIPK3 or other signaling mechanisms [52].

In platelets, necroptosis has been implicated in pathological conditions such as thrombosis [53,54], and immune-mediated thrombocytopenia [55]. While platelets are anucleate and lack transcriptional machinery, they possess functional necroptotic signaling including RIPK1, RIPK3, and MLKL, upon specific stimuli, such as inflammatory cytokines (e.g., TNF-α) or oxidative stress [52]. In platelets, TNFR1 expression is lower than in MKs, suggesting a minor role for TNFR1-induced necroptosis in this cell type [52]. In contrast, functional TLR4 is present on the platelet surface and capable of inducing MLKL-mediated necroptosis [52]. Intraperitoneal lipopolysaccharide (LPS) injection in wild-type mice reduces platelet counts by approximately 60% and this effect is absent in TLR4-deficient mice, confirming the role of TLR4-mediated necroptosis in LPS-induced thrombocytopenia [56]. Furthermore, genetic deficiency or GSK′872-mediated inhibition of RIPK3 in mice resulted in prolonged tail bleeding and delayed arterial thrombosis occlusion times [54]. These defects are associated with impaired dense delta (δ) granule release upon thrombin or thromboxane A_2_ (TXA_2_) stimulation, while α-granule release remains intact [54]. Interestingly, RIPK3 was shown to interact with Gα13, thereby indicating RIPK3-mediated Gα13 downstream signaling during thrombus growth [54]. Pharmacological inhibition of necroptosis with Necrostatin-1 or genetic deletion of MLKL could protect mice against venous thrombosis and neutrophil recruitment, highlighting the contribution of this pathway to thromboinflammation [53].

**Figure 3:**
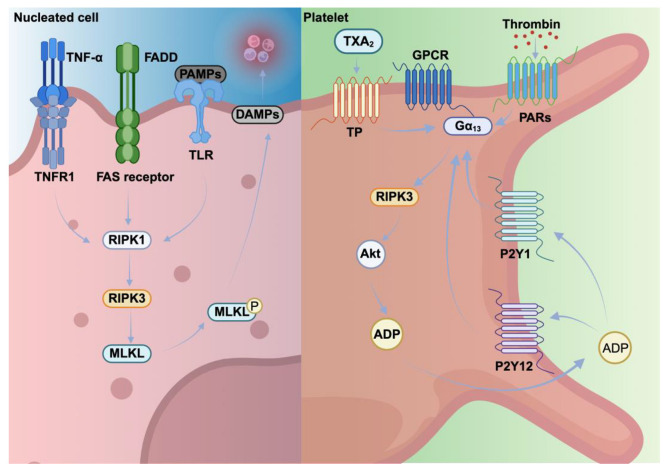
Necroptosis in nucleated cells and platelets. In nucleated cells, necroptosis is triggered when ligands like TNF-α and FasL bind receptors such as TNFR1 and TLRs. This activates RIPK3, which phosphorylates MLKL. Phosphorylated MLKL oligomerizes and moves to the plasma membrane, forming pores that release DAMPs and pro-inflammatory signals. In platelets, RIPK3 enhances TXA_2_- and thrombin-induced platelet activation by interacting with Gα13, leading to Akt activation and ADP secretion. Adenosine diphosphate (ADP), G-protein coupled receptor (GPCR), protease-activated receptors (PARs), purinergic receptors P2Y1 and 12 (P2Y1, PY12), G-protein coupled receptor (GPCR).

In summary, these findings indicate that the regulation of necroptosis in MKs and platelets differ fundamentally from nucleated cells (Figure 3, Table 3). MK viability and platelet production are unaffected by RIPK3 or MLKL deficiency. Platelet necroptosis contributes to thrombosis and thromboinflammation in cardiovascular diseases, where necroptotic platelets can enhance clot formation, thrombus growth and leukocyte recruitment. Targeting necroptotic pathways in platelets may therefore provide therapeutic benefits in conditions associated with excessive thromboinflammation and platelet activation.

## 5. Pyroptosis Pathways in Nucleated Cells Versus Platelets

### 5.1. Pyroptosis Is an Inflammatory Form of Cell Death

Pyroptosis is triggered by PAMPs or DAMPs and is characterized by distinct inflammatory responses that eliminate microbial-infected cells in pathological situations such as sepsis [57]. While pyroptotic and apoptotic cells display chromatin condensation and DNA fragmentation, pyroptosis in addition, induces cell swelling and osmotic lysis, leading to membrane rupture and the release of pro-inflammatory cytokines [58].

Pyroptosis and necroptosis also share similarities, based on the inflammatory stimuli, but the molecular components of the signaling machinery are different. In the canonical pyroptosis pathway, Nod-like receptor pyrin domain containing 1 (NLRP1) and 3 (NLRP3) inflammasomes activate caspase-1, which cleaves gasdermin D (GSDMD), thereby releasing its N-terminal domain (N-GSDMD) [59]. N-GSDMD is integrated into the plasma membrane, forming pores that release intracellular contents, including interleukin (IL)-1β and IL-18, and alarmins such as high-mobility group box 1 (HMGB1). These mediators amplify inflammation and recruit immune cells to the site of infection [59]. Additionally, a non-canonical pathway operates through caspase-4 and -5 in humans or caspase-11 in mice, which can be directly activated by LPS without requiring inflammasome assembly [60,61].

### 5.2. Pyroptotic Platelets: Drivers of Inflammatory Death

MKs and platelets express molecular components required for the pyroptosis pathway, including TLRs and Nod-like receptors (NLRs), leading to the formation of NLRP3 inflammasomes. Similarly to nucleated cells, the activation of caspase-1 and subsequent cleavage of GSDMD results in membrane pore formation [62]. Activated platelets stimulated by collagen and thrombin assembles the NLRP3 inflammasome, which regulates thrombus formation. Increased oxidative stress also activates the NLRP3 inflammasome in platelets, triggering GSDMD cleavage and the release of IL-1β [62]. ROS-mediated NLRP3 activation has been observed in platelets from patients with Crohn’s disease, immune thrombocytopenic purpura (ITP) or sepsis [62]. In murine models of sepsis, elevated levels of N-GSDMD were detected in platelets. Consequently, 50% of platelet deaths are attributed to pyroptosis in septic patients, highlighting the involvement of pyroptosis in sepsis-induced thrombocytopenia [37].

In platelets isolated from ITP, elevated NLRP3 and IL-1β correlates with reduced antioxidant capacity and the exposure of platelets to H_2_O_2_ further amplifies the expression of these pyroptotic markers, indicating a direct link between inflammasome activation and pyroptosis-induced platelet death [34]. Mice treated with LPS and nigericin showed rapid platelet swelling and membrane rupture, but these effects were absent in GSDMD-deficient platelets, suggesting the important role of platelet pyroptosis in sepsis [37]. GSDMD-dependent pyroptosis was also triggered by elevated levels of S100A8/A9, acting on the TLR4 receptor [37]. Pyroptotic platelets released oxidized mitochondrial DNA, promoting the formation of neutrophil extracellular traps (NETs), further amplifying inflammatory responses [37]. Calprotectin (S100A8/A9) is a Ca^2+^-binding protein primarily expressed in neutrophils and other myeloid cells, playing a key role in innate immunity and defense against infection. Recent studies reveal that during acute inflammation or infection, calprotectin released from neutrophils enhances pyroptosis via the TLR4/NLRP3/caspase-1/GSDMD pathway, contributing to platelet death in sepsis [37]. Platelet pyroptosis contributes to cardiovascular diseases by inducing platelet dysfunction, membrane rupture, and enhanced clearance [63].

Dengue-virus-induced NLRP3 activation and subsequent pyroptosis have been described in macrophages, monocytes and platelets. While the detailed molecular mechanism underlying Dengue-virus-mediated platelet pyroptosis remains to be fully elucidated [64,65], evidence suggests it is likely triggered by platelet binding to the virion’s envelope protein domain III [64,66].

In clinical experimental settings, cisplatin-based chemotherapy induces platelet pyroptosis. Cisplatin or etoposide activates caspase-3, which cleaves gasdermin E (GSDME) isoform, leading to N-GSDME mediated membrane pore formation [67]. The adaptor protein Flotillin-2 interacts with N-GSDME and recruits it to the plasma membrane. GSDME deficiency protects mice from cisplatin-induced platelet hyperactivity suggesting that targeting GSDME-mediated pyroptosis could reduce thrombotic risk in patients treated with chemotherapy [67]. Decrypted tissue factor (TF) forms a high-affinity complex with coagulation factor VIIa, proteolytically activating factors IX and X. This cascade results in thrombin generation, fibrin formation, and platelet activation [68]. Uncontrolled TF decryption occurs in pathological conditions, such as disseminated intravascular coagulation (DIC) or immunothrombosis. Interestingly, pyroptosis contributes to TF decryption, thereby enhancing the coagulation cascade [69].

Pharmacological inhibitors against pyroptosis have been tested in different pathological settings. MCC950 is a potent and selective inhibitor of the NLRP3 inflammasome, effectively blocking NLRP3-mediated platelet pyroptosis and hyperactivation in the cecal-ligation puncture rat model [70]. MCC950 treatment reduced caspase-1 activity and platelet aggregation in mouse models of inflammatory diseases [70]. The heme/NLRP3/Bruton’s tyrosine kinase pathway was linked to platelet pyroptosis in sickle cell disease (SCD) [71]. MCC950 has been tested clinical trials but was terminated due to hepatotoxicity. Newer derivatives are under investigation [72].

**Figure 4:**
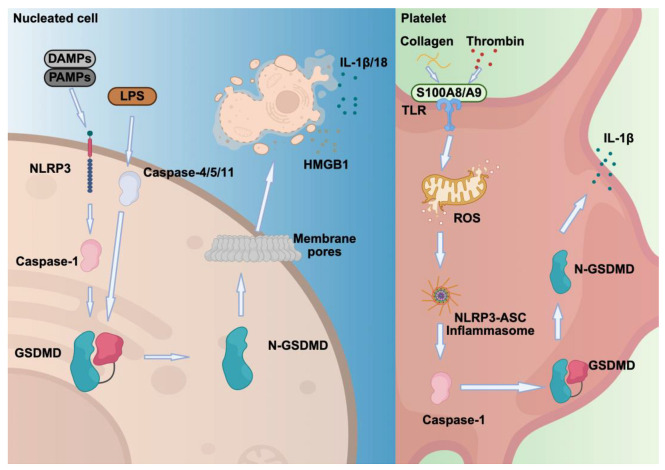
Pyroptosis in nucleated cells and platelets. Pyroptosis in nucleated cells is triggered by PAMPs or DAMPs, activating NLRP1 and NLRP3 inflammasomes. This leads to caspase-1 cleavage of GSDMD, forming pores that release inflammatory cytokines. Non-canonical pathways involve caspases 4/5 or 11, directly activated by LPS, causing cell lysis and inflammation. In platelets, stimulation by collagen and thrombin increases oxidative stress, activates inflammasomes, and triggers GSDMD-mediated pore formation, IL-1β release, and mitochondrial DNA extrusion, promoting the formation of NETs and amplifying inflammation.

In summary, pyroptosis in nucleated cells primarily serves as a defense mechanism against infections by eliminating pathogen-infected cells and releasing pro-inflammatory mediators that regulate immune cell responses (Figure 4, Table 4). In contrast pyroptosis in platelets leads to platelet dysfunction and death. Therefore, selectively targeting platelet pyroptosis without impairing immune cell pyroptosis may offer therapeutic potential to prevent thrombocytopenia in patients with viral or bacterial infections or ITPs.

## 6. Ferroptosis Pathways in Nucleated Cells Versus Platelets

### 6.1. Ferroptosis Depends on the Balance Between ROS Generation and Antioxidant Defenses

Ferroptosis is another nucleated cell death pathway, induced by iron-dependent ROS production and subsequent accumulation of lipid peroxidation products and depletion of the cellular glutathione (GSH) store [73]. Its execution involves several key molecular components associated with iron metabolism (transferrin, transferrin receptor, ferritin, ferroportin), lipid peroxidation (arachidonate lipoxygenase), and the antioxidant defense system [73]. Glutathione peroxidase 4 (GPX4) is a major component of the antioxidant defense system, preventing the accumulation of toxic lipid peroxides [73]. Therefore, GPX4 deficiency leads to ferroptosis in various cell types, highlighting its critical role in cell survival under oxidative stress. Molecular components of ferroptosis are expressed in MKs and platelets [74]. Subcellular localization of GPX4 rapidly changes during platelet activation, translocated from the cytoplasm to the plasma membrane, thereby protecting the plasma membrane from oxidative stress [74]. Consequently, reduced platelet glutathione peroxidase activity prolongs the lifespan of lipid hydroperoxides, particularly the 12-lipoxygenase product of arachidonic acid [74]. Phospholipase A2 activity is subsequently enhanced with the release of arachidonic acid, which results in higher TXA_2_ formation and platelet activation [75]. These results suggest that under pathological conditions, reduced GPX4 expression or the enzymatic activity of glutathione peroxidases contributes to abnormal platelet activation which is triggered by the accumulation of arachidonic acid and its oxidative metabolites [75]. Interestingly, in some cases, ferroptosis cannot be induced solely by GPX4 inhibition, suggesting the existence of alternative molecular mechanisms [76]. Recently, ferroptosis suppressor protein 1 (FSP1) was identified as a potent glutathione-independent inhibitor of ferroptosis, acting in parallel with GPX4 [77]. FSP1 regulates blood coagulation through a non-classical vitamin K redox cycle and effectively suppresses ferroptosis, thereby offering a potential therapeutic target in thrombotic and oxidative stress-related disorders [78].

### 6.2. Heme-Induced Ferroptosis Promotes Platelet Activation and Thrombosis

In the hematopoietic system, the pathophysiological consequences of iron overload-mediated ferroptosis were investigated by administering iron dextran into mice. Platelet count decreased after this treatment, indicating impaired platelet production [79]. In vitro differentiated MKs were treated with ferric ammonium citrate to mimic iron overload. This treatment inhibited MK differentiation and proplatelet formation, due to the activation of ferroptosis, which is monitored by mRNA expression of GPX4, ferritin heavy chain 1 (FTH1) and prostaglandin-endoperoxide synthase 2 (PTGS2), and the measurement of GSH and ROS production. The ferroptosis inhibitor ferrostatin-1 could effectively protect MKs from cellular damage [79].

In iron-overload conditions, ferroptosis not only drives tissue damage but may also promote platelet activation through iron- and ROS-mediated signaling. This contributes to a prothrombotic and pro-inflammatory state observed clinically, such as in vaso-occlusive crises in SCD [80]. Therefore, targeting ferroptosis pathways may offer therapeutic benefits by reducing aberrant platelet activation and improving vascular outcomes in these disorders. Interestingly, hemochromatosis with iron overload does not cause thrombocytopenia in humans. Platelet function remains normal in patients with hemochromatosis [81], suggesting an adaptive regulatory mechanism that protect MKs and platelets from ferroptosis [81,82]. However, rapid iron overload can increase ROS production and disrupt multiple metabolic pathways in platelets [83]. The elevated labile iron pool in the cytoplasm affects the arachidonic acid and TXA_2_ metabolic cascade and associated signaling. Conversely, iron chelators such as deferoxamine effectively inhibit platelet aggregation, TXA_2_ synthesis, and both cyclooxygenase and lipoxygenase activities [84]. These findings suggest that iron-induced ferroptosis probably impacts platelet production and function in pathological conditions in which excessive iron levels are increased rapidly within a short time. During hemolysis or rhabdomyolysis, large amounts of iron and heme (hemin) accumulate locally, thereby modulating pathological signals of ferroptosis. Under healthy conditions, hemoglobin and heme (hemin) are rapidly captured by haptoglobin and hemopexin in the blood to prevent cytotoxicity [85]. In hemolytic disorders, platelets become hyperactive, leading to microvascular thrombosis and blood clot formation in vital organs such as lungs and kidneys. Red blood cells can undergo eryptosis, releasing free hemoglobin that degrades into heme and further oxidized to hemin [85]. While a moderate increase in hemin levels supports local hemostasis, abnormally elevated hemin levels under hemolysis activates platelets, induces endothelial damage and subsequent thrombosis, and dysregulates the coagulation cascade. Recently, hemin was identified as an activator for platelet GPVI and C-type lectin-like receptor-2 (CLEC-2) signaling [85,86,87]. Protoporphyrin-IX, which shares a similar structure to hemin but lacks iron binding, inhibits CLEC-2-dependent platelet activation, suggesting that activation is iron-dependent rather than driven by the porphyrin ring [88]. In line with this, iron alone can activate platelet CLEC-2 signaling [88]. Hemin-dependent platelet activation and thrombus formation are also regulated by cyclic guanosine monophosphate-cyclic guanosine monophosphate kinase I (cGMP-cGKI) signaling, generating distinct platelet subpopulations, increasing ROS levels, and mainly targeting metabolic products of arachidonic acids [89]. Modulating cGMP levels via the cGMP-cGKI axis could reduce hemin-induced ferroptosis [89].

Conventional antiplatelet therapies fail to prevent patients from hemin-induced platelet activation and thrombosis; therefore, alternative strategies need to be developed. Recently, a C-X-C chemokine receptor type 7 (CXCR7) agonist has been proposed to inhibit hemin-induced procoagulant platelet formation and microvesiculation in mice [89]. Whether hemin may inhibit negative regulatory components of the complement (coagulation) systems, thereby attenuating plasma factor-mediated CXCR7 receptor activation, remains to be investigated. Interestingly, unconjugated bilirubin (UCB) protects platelets against hemin-induced ferroptosis by reducing ROS levels and lipid peroxidation [90]. UCB blocks the hemin-CLEC-2 interaction, thereby inhibiting downstream effector function of Syk kinase [90]. Of note, jaundice typically occurs in human neonates, due to unconjugated hyperbilirubinemia, where elevated UCB crosses the blood–brain barrier, which is a risk factor for developing encephalopathy or kernicterus [91]. Although UCB has a potent antioxidant and therapeutic potential, it has a dual protective and toxic nature for the nervous system [92].

Melatonin (MLT) is essential for regulating circadian rhythms. Interestingly, MLT has antioxidant, anti-inflammatory, and cytoprotective functions. MLT inhibits platelet aggregation induced by ADP, thrombin and arachidonic acid [93]. Activation of the MLT receptor with agomelatine effectively reduces platelet aggregation in response to arachidonic acid, indicating antithrombotic potential in melatonin-induced signaling [94]. Interestingly, hemin-induced ferroptosis is attenuated by MLT in platelets [95]. This protective effect occurs via MLT activation, restoring mitochondrial function and glutathione balance by suppressing hemin-induced ROS production and lipid peroxidation [94].

**Figure 5:**
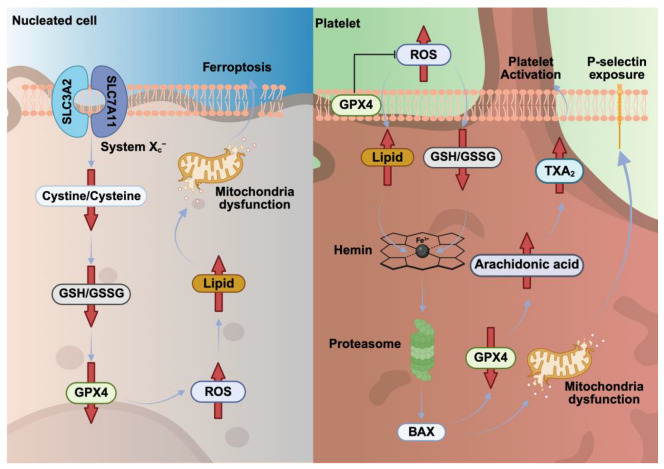
Ferroptosis in nucleated cells and platelets. Ferroptosis in nucleated cells results from reduced System Xc− activity and GPX4 function, causing GSH depletion and lipid ROS accumulation, which causes oxidative damage. In platelets, iron-dependent ROS cause lipid peroxidation and GSH depletion, increasing arachidonic acid release, TXA_2_ production, and platelet activation. During this process, GPX4 translocates to the plasma membrane to help protect against oxidative stress. Glutathione/oxidized glutathione (GSH/GSSG).

In summary, in nucleated cells, ferroptosis is an iron-dependent form of cell death driven by ROS generation, lipid peroxidation, and GSH depletion, with GPX4 acting as a key antioxidant defense (Figure 5, Table 5). In platelets, GPX4 translocates to the membrane during activation, protecting against oxidative stress, while reduced GPX4 activity prolongs the lipid peroxide lifespan, enhancing platelet activation via arachidonic acid metabolism. Iron overload or heme release during hemolysis can induce ferroptosis in MKs, impairing platelet production and promoting platelet activation and thrombosis through iron- and ROS-mediated signaling.

## 7. Conclusions

Beyond traditional apoptosis and necrosis, the discovery of novel cell death pathways such as pyroptosis and ferroptosis in nucleated cells has expanded our understanding of platelet pathobiology. Molecular components of nucleated cell death pathways are expressed in MKs and platelets and involved in platelet production and function. Activated cell death pathways strongly influence energy metabolism, antioxidant defense, and ROS production, which significantly affect platelet receptor functions and their signaling networks, mainly regulating Ca^2+^-dependent procoagulant platelet activity and platelet lifespan. Accordingly, compelling experimental and clinical evidence indicates converging pathomechanisms and cross-talk among the indicated cell death pathways in different disease conditions (Table 6). The interplay between diverse cell death pathways reflects a complex but highly coordinated response to pathological stress. Ca^2+^ signaling and ROS emerge as common mediators of this crosstalk. Ca^2+^ channels, including transient receptor potential channels (TRPs), the store-operated calcium entry (SOCE) channel ORAI, mechanosensitive channel Piezo, and purinergic receptor 7 (P2X7), effectively modulate Ca^2+^ dynamics, which in turn, regulate mitochondrial integrity, inflammasome activation, and ROS-induced lipid peroxidation.

Intracellular ROS levels are increased by mitochondrial dysfunction and iron and Ca overload, regulating signaling molecules, as well as channel and enzymatic activities. Consequently, both nucleated cells and platelets expose PS on the cell surface and release granule contents and microvesicles. In disease conditions such as sepsis, SCD, cardiomyopathy, atherosclerosis, and stroke, parallel activation of cell death pathways has been detected (Table 6). These pathways intersect and amplify one another, contributing to inflammation, tissue injury, and organ dysfunction. This integrated network of cell death underlies key pathological features such as immunoparalysis in sepsis, thrombosis in SCD, atherosclerosis, and neurovascular collapse in stroke. Understanding the molecular crosstalk between cell death pathways, particularly the roles of Ca^2+^ channels and ROS, offers promising new targets for therapeutic intervention, restoring cellular homeostasis.

Although several reports discuss the pathological roles of cell death pathways in MKs and platelets, future studies need to be performed to dissect the role of molecular components of platelet cell death pathways in immunothrombosis, thromboinflammation and different platelet-related disorders. Further investigations may advance our knowledge to develop a new strategy against aberrant blood clotting, thrombocytopenia, and thrombotic complications, triggered by the indicated cell death pathways.

## Figures and Tables

**Table 1:** Comparison of apoptosis in nucleated cells and platelets.

	Nucleated Cells		Platelets
Triggering factors	Death receptors	Cellular stress factors	Do not express FAS or TRAIL receptors
Pathways	Extrinsic	Intrinsic	Intrinsic
Triggering mechanism	Ligand binding → adaptor proteins → caspases	Caspases 8 → BID → BAX/BAK → Mitochondrial damage	BAX/BAK → Mitochondrial damage
Key regulatory proteins	Caspases 3/6/7, BID		BCL-XL, BAX, BAK
Morphological signs	Cell shrinkage, DNA fragmentation, chromatin condensation, membrane blebbing and PS exposure		PS exposure and membrane blebbing
Outcomes	Cell death		Procoagulant activity (BH3-only proteins–mimetism) Severe thrombocytopenia (BCL-XL deficiency) Prolonged platelet lifespan (BAD, BAK deficiency)

**Table 2:** Comparison of necrosis in nucleated cells and platelets.

	Nucleated Cells	Platelets
Triggering factors	Ischemia, trauma and infection	Mechanical stress and high ROS
Pathways	Ca^2+^ ↑ → MPTP formation → Δψm collapse → ROS increase →	Ca^2+^ ↑ → MPTP formation via MCU and CypD → PS exposure
Morphological changes	Membrane rupture, cytoplasmic disintegration and nuclear fragmentation	Platelet swelling and fragmentation
Key regulatory proteins	CypD, components of MPTP	Cyclophilin D, MCU
Outcomes	Extensive tissue inflammation and damage	Coagulation and clot stabilization

**Table 3:** Comparison of necroptosis in nucleated cells and platelets.

	Nucleated Cells	Platelets
Triggering factors	TNF-α, PAMPs, TLR activation	TNF-α, oxidative stress, TLR4 activation, thrombosis
Pathways	TNFR1 or TLR activation → RIPK1→ RIPK3 → MLKL→ Membrane pore formation	TLR4 activation → RIPK1 → RIPK3 → MLKL
Morphological changes	Plasma membrane swelling, organelle swelling, rupture of plasma membrane, nuclear fragmentation and cellular lysis	Platelet activation and granule release
Key regulatory proteins	RIPK1, RIPK3, MLKL	RIPK3, MLKL
Outcomes	Cell lysis, DAMPs, and cytokines release and tissue damage	Clot formation, thrombosis and thromboinflammation

**Table 4:** Comparison of pyroptosis in nucleated cells and platelets.

	Nucleated Cells	Platelets
Triggering factors	PAMPs or DAMPs	PAMPs, DAMPs, oxidative stress and microbial infections
Pathways	Canonical: NLRP3 → Caspase-1 → GSDMD → Membrane Pores	NLRP3 → Caspase-1 → GSDMD → membrane pores
	Non-canonical: Caspase-4/5 (or Caspase-11 in mice) →LPS activation	
Morphological changes	Cell swelling, chromatin condensation and membrane rupture	Platelet swelling, membrane pore formation and IL-1β release
Key regulatory proteins	NLRP3, caspase-1, GSDMD, IL-1β, IL-18	NLRP3, caspase-1, GSDMD, TLR4, S100A8/A9
Outcomes	Cytokine release (IL-1β, IL-18) and immune cell recruitment	Thrombosis and inflammation

**Table 5:** Comparison of ferroptosis in nucleated cells and platelets.

	Nucleated Cells	Platelets
Triggering factors	Iron-dependent ROS production, neurodegeneration and cancer	Iron overload (e.g., heme/hemin), hemolysis and rhabdomyolysis
Pathways	ROS → Fe^2+^ ↑ → Lipid peroxidation ↑→ GSH depletion → GPX4 inactivation → Ferroptosis	Eryptosis → Hemin → Fe^2+^ → ROS ↑ → Lipid peroxidation ↑ → GSH depletion ↓ → GPX4 translocation → TXA_2_ ↑ → Platelet activation
Molecular components	Transferrin, ferritin, ferroportin, GPX4, FSP1	Transferrin, ferritin, GPX4, FSP1, GSDMD, hemin, S100A8/A9, TLR4
Morphological changes	Lipid peroxidation → Membrane rupture → Cell death	Lipid peroxidation → Membrane pore→ Platelet swelling and activation
Outcomes	Cell death accompanied with DAMPs and cytokine release	Platelet activation and aggregation, cytokine release and thrombosis

**Table 6:** Mechanistic intersections of cell death pathways in various pathologies.

Disease Context	Cell Death Pathways	Calcium Channels	ROS Production	Crosstalk
**Sepsis/** **Systemic Inflammation**	Apoptosis, Necroptosis Pyroptosis Ferroptosis	TRPM2 [96] CICR [97] SOCE [98,99]	Activation of NLRP3 complex; Mitochondrial damage and cytokine /ROS release; Accumulation of free iron enhances ROS production, lipid peroxidation.	Pyroptosis drives inflammation. Necroptosis, ferroptosis and apoptosis occur in parallel, triggering multiorgan failure (coagulopathy, microthrombus formation, endothelial dysfunction, immunoparalysis) [100].
**Sickle Cell Disease (SCD)**	Ferroptosis Pyroptosis Necrosis	Piezo TRPs [101,102]	Hemolysis-associated ROS production; ROS-mediated thrombosis, abnormal red blood cell and platelet adhesion and lipid peroxidation.	Heme-mediated NLRP3 activation and pyroptosis. Ferroptosis is driven in parallel by lipid ROS and iron overload [103].
**Iron Overload/** **Cardiomyopathy**	Ferroptosis Apoptosis	LTCC [104]	Iron overload-induced ROS production triggering both apoptosis and ferroptosis.	Iron overload-mediated lipid peroxidation and ferroptosis. Mitochondrial ROS-induced endothelial dysfunction and apoptosis. Both pathways converge on ROS [105].
**Atherosclerosis/** **Thrombosis**	Apoptosis Pyroptosis Necroptosis	TRPs [106] P2X7 [107] SOCE [108]	Endothelial dysfunction and inflammasome activation and foam cell death	Apoptosis-mediated endothelial damage; endothelial and macrophage apoptosis destabilizing necrotic plaques; pyroptotic macrophages triggers vascular inflammation; necroptosis-induced plaque rupture and thrombosis, monocyte and platelet adhesion [109].
**Stroke/Ischemia–Reperfusion Injury**	Necrosis Apoptosis Ferroptosis Necroptosis	SOCE [110] TRPs [111,112] P2X7 [113]	Lipid peroxidation	Pyroptosis-mediated inflammatory cell death; blood–brain barrier rupture; Intracerebral hemorrhage-mediated hemolysis triggering ferroptosis and necroptosis; Necroptosis-mediated neurodegeneration; Intracranial mechanical force-induced necrosis; Mitochondrial Ca^2+^ overload-induced apoptosis and necrosis. ROS and iron accumulation triggering ferroptosis [114].

Transient receptor potential cation channel, subfamily M, member 2 (TRPM2); calcium-induced calcium release (CICR); store-operated calcium entry (SOCE); transient receptor potential channels (TRPs); L-type calcium channel (LTCC).

## Data Availability

No new data were created or analyzed in this study.

## Abbreviations

The following abbreviations are used in this manuscript:

ADP	Adenosine diphosphate
ANT	Adenine nucleotide translocator
ATP	Adenosine triphosphate
BAK	BCL-2 homologous antagonist/killer
BAX	BCL-2-associated X protein
BCL-2	B-cell lymphoma 2
BCL-XL	B-cell lymphoma-extra large
BID	BH3 interacting-domain death agonist
Ca^2+^	Calcium
cGMP-cGKI	Guanosine monophosphate-cyclic guanosine monophosphate kinase I
CLEC-2	C-type lectin-like receptor-2
CXCR7	C-X-C chemokine receptor type 7
CypD	Cyclophilin D
DAMPs	Damage-associated molecular patterns
DIC	Disseminated intravascular coagulation
DISC	Death-inducing signaling complex
DNA	Deoxyribonucleic acid
DR4	Death receptor 4
DR5	Death receptor 5
FADD	Fas-associated death domain
Fas CD45	Cluster of differentiation CD45
FasL	Fas ligand
FSP1	Ferroptosis suppressor protein 1
FTH1	Ferritin heavy chain 1
GPCR	G-protein-coupled receptor
GPVI	Glycoprotein VI
GPX4	Glutathione peroxidase 4
GSDMD	Gasdermin D
GSDME	Gasdermin E
GSH	Glutathione
GSH/GSSG	Glutathione/oxidized glutathione
HMGB1	High-mobility group box 1
IL-18	Interleukine 18
IL-1β	Interleukine 1 beta
ITP	Immune thrombocytopenic purpura
LPS	Lipopolysaccharides
LTCC	L-type calcium channel
MCU	Mitochondrial calcium uniporter
MKs	Megakaryocytes
MLKL	Mixed lineage kinase domain-like protein
MLT	Melatonin
MPTP	Mitochondrial permeability transition pore
NETs	Neutrophil extracellular traps
N-GSDMD	N-terminal domain of GSDMD
NLRP1	Nod-like receptor pyrin domain containing 1
NLRP3	Nod-like receptor pyrin domain containing 3
NLRs	Nod-like receptors
OSCP	Oligomycin-sensitive binding protein
P2Y1	Purinergic receptor P2Y1
P2Y12	Purinergic receptor P2Y12
PAMPs	Pathogen-associated molecular patterns
PARs	Protease-activated receptors
PiC	Mitochondrial inorganic phosphate carrier
PS	Phosphatidylserine
PTGS2	Prostaglandin-endoperoxide synthase 2
RIPK1	Receptor-interacting protein kinase 1
RIPK3	Receptor-interacting protein kinase 3
ROS	Reactive oxygen species
SCD	Sickle cell disease
SMAC	Second mitochondria-derived activator of caspases
SOCE	Store-operated calcium entry
TF	Tissue factor
TLRs	Toll-like receptors
TNFR1	Tumor necrosis factor receptor
TNF-α	Tumor necrosis alpha
TRAIL	TNF-related apoptosis-inducing ligand
TRPM2	Transient receptor potential cation channel, subfamily M, member 2
TRPs	Transient receptor potential channels
TXA_2_	Thromboxane A_2_
UCB	Unconjugated bilirubin
VDAC	Voltage-dependent anion channel
